# Green microwave synthesis of ZnO and CeO_2_ nanorods for infectious diseases control and biomedical applications

**DOI:** 10.1186/s13568-022-01495-7

**Published:** 2022-12-12

**Authors:** Nevein Gharbia, Sabha Elsabbagh, Ali Saleh, Hoda Hafez

**Affiliations:** 1 Environmental Studies and Research Institute (ESRI), University of Sadat City, Sadat City, 23897 Menofia Egypt; 2 Department of Microbiology, Faculty of Science, Menofia University, Al Minufiyah, Menofia Egypt; 3 Environmental Geology Lab. Survey of Natural Resources, Environmental Studies and Research Institute (ESRI), University of Sadat City, Sadat City, 23897 Menofia Egypt; 4 Nano Photochemistry Laboratory, Nanotech. Dept., Environmental Studies and Research Institute (ESRI), University of Sadat City, Sadat City, 32987 Menofia Egypt

**Keywords:** CeO_2_, ZnO nanorods, Microwave-assisted green synthesis, Infectious diseases control, Biomedical applications

## Abstract

Control of Infectious diseases such as; bacteria and viruses, has become a globally critical issue, since the appearance of COVID-19 virus in 2020. In addition to the microbial resistance of the currently available therapeutic drugs as well as, its prolonged side effects make its use is of health care concern. Green nanotechnology approach is a promising solution for controlling such infectious diseases and many biomedical purposes. In the present study, green synthesis approach based on microwave-assisted hydrothermal method is an innovative and environmentally friendly method for preparation of bioactive CeO_2_ and ZnO nanorod structured materials using *Olea europaea (O. e.)* leaf plant extract as a natural medicinal capping agent for controlling the shape and size of nano-products. The optical and structural analyses of the obtained nanorod-structures are characterized using; TEM, FTIR, XRD, SBET analyses and particle size analyzer. The green-synthesized ZnO and CeO_2_ nanorods display an average crystallite size of approximately 15 and 5 nm, respectively. The antimicrobial activity of ZnO and CeO_2_ nanorods compared with the traditional hydrothermal methods, was examined on six clinical pathogens including; (*E. coli Serratia *sp.*, S. aureus, Bacillus subtilis, Streptococcus mutant, and MRSA*). The results indicated superior antimicrobial and anti-tumor activities towards hepatocellular carcinoma cell lines (IC_50_ = 117.24 and 103.50 μg mL^−1^ for ZnO and CeO_2_ and LD_50_ > 3000 mg kg^−1^). This demonstrates that the green microwave process is a promising approach for the synthesis of effective ZnO and CeO_2_ nanomaterials applied for many biomedical applications.

**Supplementary Information:**

The online version contains supplementary material available at 10.1186/s13568-022-01495-7.

## Key points

- Green synthesized ZnO and CeO_2_ nanorods.
- High antimicrobial activity against gram-negative and gram-positive bacteria.
- Non-toxic and antitumor activity against hepatocellular carcinoma cell lines.

**Supplementary Information:**

The online version contains supplementary material available at 10.1186/s13568-022-01495-7.

## Introduction

New and emerging infectious diseases such as COVID-19 and many other microbial diseases pose an increasing global health threat and complicate the health situation all over the world. These diseases will lead to the development of a major problem that affects the economy and the productivity of individuals. Diseases often occur when ambient conditions deteriorate and lead to heavy losses. Many microbes have developed resistance to specific antibiotic treatments as a result of the extensive and frequently indiscriminate use of antimicrobial medications, and these strains are particularly noticeable in the hospital setting. Treating these infectious diseases, has led to a significant clinical problem (Tang et al. 2020; Aljibali et al. 2022). Additionally, cancer is a severe health issue and one of the main killers in the world. Treatment for cancer with various anti-cancer medications is based on biological, anti-metabolite, and alkylating substances, etc. One of the biggest issues with these medications is the toxicity that results from the inability of cancer cells to differentiate from normal cells, which causes systemic toxicity (Wiesmann et al. 2020). Green nanotechnology is a cutting-edge technology today with numerous potential applications in the environmental, industrial, medicinal, etc. Green nanotechnology is based on the use of natural resources, the elimination of hazardous solvents and energy-saving processes to produce safe and eco-friendly nanomaterials (Charbgoo et al. 2017; Alvaredo et al. 2014). Nanomaterials have been synthesized using variation of chemical and physical procedures, including electrospinning, lithography, and sputtering (Gonçalves et al. 2021).

However, these conventional synthesis methods are quite expensive and may result in formation of toxic by-products that cause health risks. Green synthesis approach is a smart and innovative solution to overcome the risk effect of synthetic nanomaterials on health and environment. As a result, the majority of current research has focused on quick and environmentally friendly ways for production of nanomaterials (Santhoshkumar et al. 2014; Faisal et al. 2021; Naidi et al. 2021; Tran et al. 2022).

Plant extracts as more suitable alternatives to traditional chemicals used for synthesis of nanomaterials as a green approach. In green synthesis method, plant extracts replaces the chemical reagents for conventional processes by phyto-constituents presentiments in the plant parts such as flavonoids, polyphenols, carbohydrates, etc. not only as stabilizing, capping and reducing agents, assist in controlled morphology of nanoparticles (Pujar et al. 2021).

In this work, biological components from *Olea europaea* leaf extract were used to produce ZnO and CeO_2_. *Olea europaea* Belongs to the Oleaceae family and makes up over 98% of the world's harvest. It is one of the most important fruit trees grown in the Mediterranean region. Due to their nutritional value and health advantages, olive leaves are an agricultural byproduct that has gained increasing attention from the scientific and industrial communities. *Olea europaea* leaves were therefore seen as prospective sources of bioactive substances, particularly phenolic compounds. Oleuropein, Hydroxytyrosol, and other flavonoids like Apigenin-7-glucoside, Luteolin-7-glucoside, and Verbascoside are the most prevalent abundant substances. (Sellami et al. 2021).

In this research, The use of *Olea europaea* leaf plant extract as a reducing and stabilizing agent during the microwave-assisted synthesis of ZnO and CeO_2_ nanoparticles is an environmentally friendly method because it is quick, safe, economical, and does not need the use of chemical solvents. The morphological and structural analyses of the green synthesized metal oxide nanomaterials have been studied. Compared with the traditional hydrothermal methods, the antimicrobial, as well as the antitumor activity of the green synthesized nanomaterials, are examined. As this is the first time to make a comparison between green-synthesized ZnO and CeO_2_ nanorods with the nanoparticles synthesized via traditional hydrothermal methods.

## Material and methods

### Materials

For the seeding of ZnO, zinc acetate and sodium hydroxide (NaOH) were supplied from lobacheme. For the production of CeO_2_, cerium nitrate Ce (NO_3_)_3_.6H_2_O was bought from Sigma Aldrich. Merck provided the Dimethylsulfoxide (DMSO) and nutrient agar media that were utilized to determine Zone of Inhibition (ZOI). All compounds were analytical grade and require no further purification.

### Microwave digestion device

Microwave digestion Speed Wave XPERT device from Berghof, Germany, was used for synthesis of ZnO and CeO_2_ nanorod structures. The Magnetron power 1000 W with maximum temp. up to 260 °C and pressure 100 bar. The device was created by the Berghof Fluoroplastic Technology GmbH using eight 100 mL Teflon vessels with the highest quality standards. They are made of premium TFM-PTFE and impressed through dependability and an outstanding lifespan.

### Preparation of *Olea europaea* leaf plant extract.

Fresh and healthy leaves of *Olea europaea (O. e.)* were washed multiple times with deionized water to get rid of the dust particles on their surface then they were dried in shade.

A healthy and undamaged leaf was carefully chopped, and stirred with deionized water at 85 °C for 2 h. The extract was filtered through Whatman filter paper after it was allowed to cool. The filtered leaf extract was used for further experiments as, capping agent, stabilizer and reducing agent for synthesis of the metal oxides nanomaterials, being usable within 2 weeks. The storage done in Argon atmosphere. (Chatterjee et al. 2016; Rosi et al. 2018).

### Synthesis of ZnO nanomaterials

ZnO nanorods (NRs) are prepared via environmentally friendly and fast microwave-assisted green hydrothermal method. As in a normal procedure, 12 gm of Zinc acetate hydrated was dissolved in 100 mL of distilled water to form a transparent solution. 15 mL Sodium hydroxide (NaOH) (5Molar) was added to the above solution gradually and under vigorous stirring. After formation of Zn (OH)_4_ precipitate, it was filtered, cleaned with distilled water, and allowed to dry at room temperature. Then suitable amount from the dried powder was added to 50 mL of (*O. e.*) extract and/or 50 mL of bi-distilled water. The suspension was put under continuous stirring and sonication for 30 min at room temperature to form a homogeneous suspension. The suspension was then transferred either into four Berghof TFM-PTFE microwave Teflon vessels at 160 °C for 30 min, or for comparison, into 100 mL Teflon-lined stainless-steel autoclave at 160 °C for 48 h, as a traditional hydrothermal synthesis method. After that, the Teflon vessels were allowed to cool down naturally and the resultant nanoproduct was harvested by centrifugation and washed with distilled water, and finally dried at 60 °C in the oven and then milled in a porcelain mill (Abdelmordy 2017).

### Synthesis of CeO_2_ nanomaterials

4 gm of cerium nitrate hexahydrate was dissolved in 100 mL of distilled water to form a transparent solution. Sodium hydroxide (NaOH) was added to the above solution gradually and under vigorous stirring. After formation of Ce (OH)_4_ precipitate, it was filtered, cleaned with distilled water, and allowed to dry at room temperature.

Then suitable amount of the dried powder was added to 50 mL of (*O. e.* L.) extract and/or 50 mL of bi-distilled water. The suspension was put under continuous stirring and sonication for 30 min at room temperature to form a homogeneous suspension. The suspension was then transferred either into four Berghof TFM-PTFE microwave Teflon vessels at 200 °C for 30 min, or for comparison, into 100 mL Teflon-lined stainless-steel autoclave at 200 °C for 12 h, as a traditional hydrothermal synthesis method. The Teflon vessels were then allowed to cool naturally, and the last product was obtained by centrifugation, washed with distilled water, and then dried at 60 °C in the oven before being milled in a porcelain mortar (Maqbool 2017).

### Characterization

X'pert Philips X-ray diffraction (XRD) Using Cu K radiation, 40 kV, 30 mA, and a scan rate of 50/min, was used to examine the surface characteristics of ZnO and CeO_2_ nanomaterials. Utilizing Scherer's formula, the crystallite size of the produced materials was calculated from the broadening of the relevant X-ray peaks (Wang et al. 2020). The morphology of the produced nanomaterials was determined by using a transmission electron microscope (TEM), JEM-2000 EX (JEOL, Tokyo, Japan). Brunauer–Emmett–Teller (BET) analysis (Quantachrome Instruments, NOVA series, USA) was used to calculate the specific surface area (m^2^ g^−1^) and pore volume of the powders at 77.35 K. A multipoint BET technique was used to calculate the specific surface area (SBET) using adsorption data at relative pressures (P/P0) between 0.05 and 0.25. The Barrett-Joyner-Halenda (BJH) method was utilized to determine the pore size distribution using the adsorption–desorption isotherm. The pore volume and average pore size were calculated using the nitrogen adsorption volume at the relative pressure (P/P_0_) of 0.976.

The Zeta Sizer Nano (ZS), a high performance two angles particle size, zeta potential, and molecular weight analyzer ideal for measurement of small volume samples at very low or high concentration and the detection of aggregates, was used to calculate the average particle size of synthesised nanomaterials using the Dynamic Light Scattering (DLS) technique. Non-Invasive Backscatter Optics (NIBS) performs significantly better than 90 scattering optics systems (Lopez et al. 2016).

The chemical composition and quality of the synthesized ZnO and CeO_2_ nanomaterials were evaluated using FTIR 430 spectrometer (Jasco-Japan) equipped with a diffuse reflectance accessory (Harrick, USA) in the range of 400–4000 cm^−1^.

### Anti-bacterial activity assay

The antibacterial activity assay of the green synthesized ZnO and CeO_2_ nanomaterials compared with that prepared via traditional hydrothermal methods, has been studied against six carefully chosen bacterial strains; two-gram negative bacteria, i.e. *E. coli (ATCC 8739)* and *Serratia marcescens* brought from faculty of Pharmacy, Tanta University, Egypt and four-gram positive bacteria, i.e. *Staphyllococcus aureus* (ATCC 6538), *Bacillus subtilis (ATCC 6633*), *Streptococcus mutant (ATCC 25175),* and *Methicillin-Resistant Staphylococcus Aureus (MRSA)* obtained from faculty of Aquatic and fisheries sciences-Kafr elsheikh university. The experiment is carried out in DMSO solvent using the agar diffusion method (Nabi et al. 2020). The Zone of Inhibition (ZOI) was determined by using Kirby–Bauer disc diffusion susceptibility technique (Ningappa et al. 2008). The experiment was carried out in accordance with the guidelines established by the National Committee for Clinical Laboratory Standards (NCCLS, 1999).The pathogens under investigation were grown in 10 cm test plates on nutrient agar. The test plates were incubated for 48 h at 37 °C. The zone of inhibition (in mm diameter) was observed and recorded after the incubation period. Three different types of assays were carried out to study the antimicorbial activity of the green-synthesized NPs in the concentration range of 50–200 µg mL^1^. This is in order to determine the most active conc. towards the bacterial strains under investigation. 50 µg mL^−1^ was chosen as the MIC that gives a remarkable antimicrobial effect.

The experiments were carried out in triplicate, and the results are given as means with standard deviations (SD) for three parallel measurements. Antibiotics amoxicillin and flucloxacillin were used as controls. DMSO was used as negative control.

### In-vitro antitumor cytotoxicity assay

The antitumor activity of the microwave-assisted green synthesized ZnO and CeO_2_ nanorods has been examined on hepatocellular carcinoma cell lines, according to van de Loosdrecht et al. (1994). The cell viability was determined by using MTT assay method. In a typical method, hepatocellular carcinoma cell line was seeded in a plate of 96 wells plate (1 × 10^5^ cells/well) and incubated for 1–5 h at 37 °C in a 5% CO_2_ incubator to allow cell MTT to be metabolized. After incubation, the media were replaced with new fresh media treated with serial concentrations of ZnO and/or CeO_2_ nanomaterials (31.25 to 1000 µg mL^−1^). After incubation, Formazan (MTT metabolic product) was resuspended in 200ul DMSO and was shaken at 150 rpm for 5 min to thoroughly mix the formazan into the solvent. The optical density was measured at 560 nm and background was subtracted at 620 nm. Optical density should be proportional to cell quantity. The IC_50_ values were determined using nonlinear regression analysis with log (inhibitor) vs. variable slope (four parameters) (Van de Loosdrecht 1994).

### In-vivo antitumor toxicity assay

The studied sample was administered orally to clusters of mice, every consisting of six animals, in gradually increasing dosages ranging from 0.1 to 3 g kg^−1^, in accordance with the procedure outlined by Finny (1964). The identical conditions were used to receive and care for the control animals. For 1 h, animals were continuously checked for abnormal characteristics. After administration, each group's mortality rate was tracked for 24 h (Finny 1964; El-Shafey et al. 2020).

## Result

### Morphological analysis

**Fig. 1:**
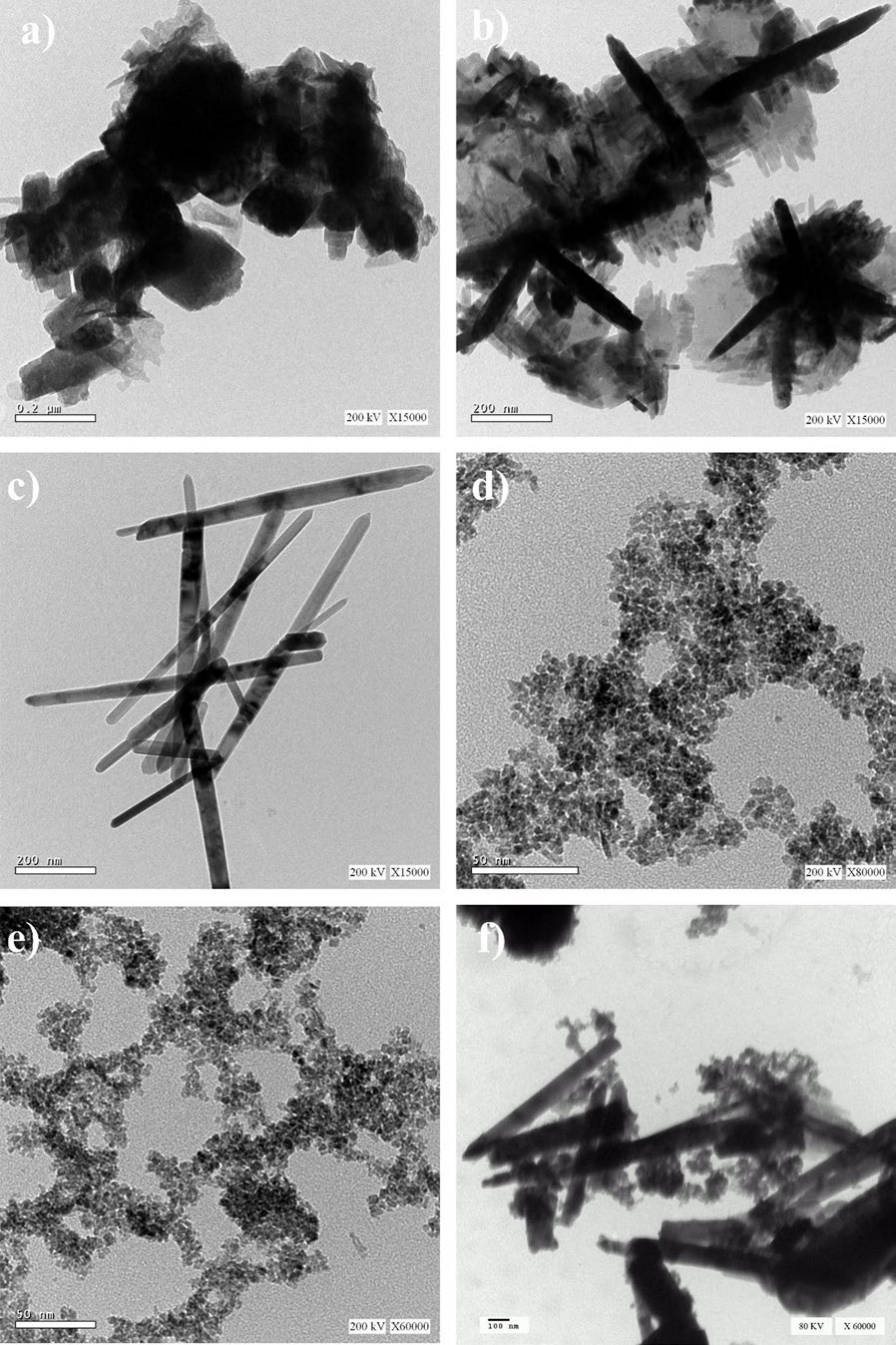
TEM images of ZnO and CeO_2_ nanoproducts prepared via microwave-assisted hydrothermal methods compared with traditional hydrothermal methods in the presence and absence of *Olea* leaf extract as a green capping agent

The surface morphology of ZnO and CeO_2_ nanomaterials is investigated by TEM analysis (Fig. 1a). The TEM image of ZnO nanoparticles prepared by traditional hydrothermal methods (Fig. 1a), shows ZnO nanosheets with a high transparency. The dark textures in the TEM image are attributed to overlapping section of nanosheets. This is in consistent with the results of Xiaoyan et al. (2014). By using microwave hydrothermal methods without addition of *(O. e.)* extract, nanorod/nanosheet heterostructure from ZnO is formed (Fig. 1b). However, by addition of *(O. e.)* extract during the green microwave synthesis, well-defined ZnO nanorod structure is observed (Fig. 1c) with an average diameter (40–60 nm) and length (400–700 nm).

The TEM images of CeO_2_ nanoparticles prepared either by traditional hydrothermal methods (Fig. 1d, e) or by microwave hydrothermal methods show homogeneous nanoparticles with spherical morphology with an average size from 3 to 6 nm. However, by adding *O.e* leaf plant extract as a capping agent leads to controlling the shape of the CeO_2_ nanoproducts and produces rod-like shape morphology (Fig. 1f) with an average diameter (29–37 nm) and length (200–500 nm).

### Crystallographic analysis

**Fig. 2:**
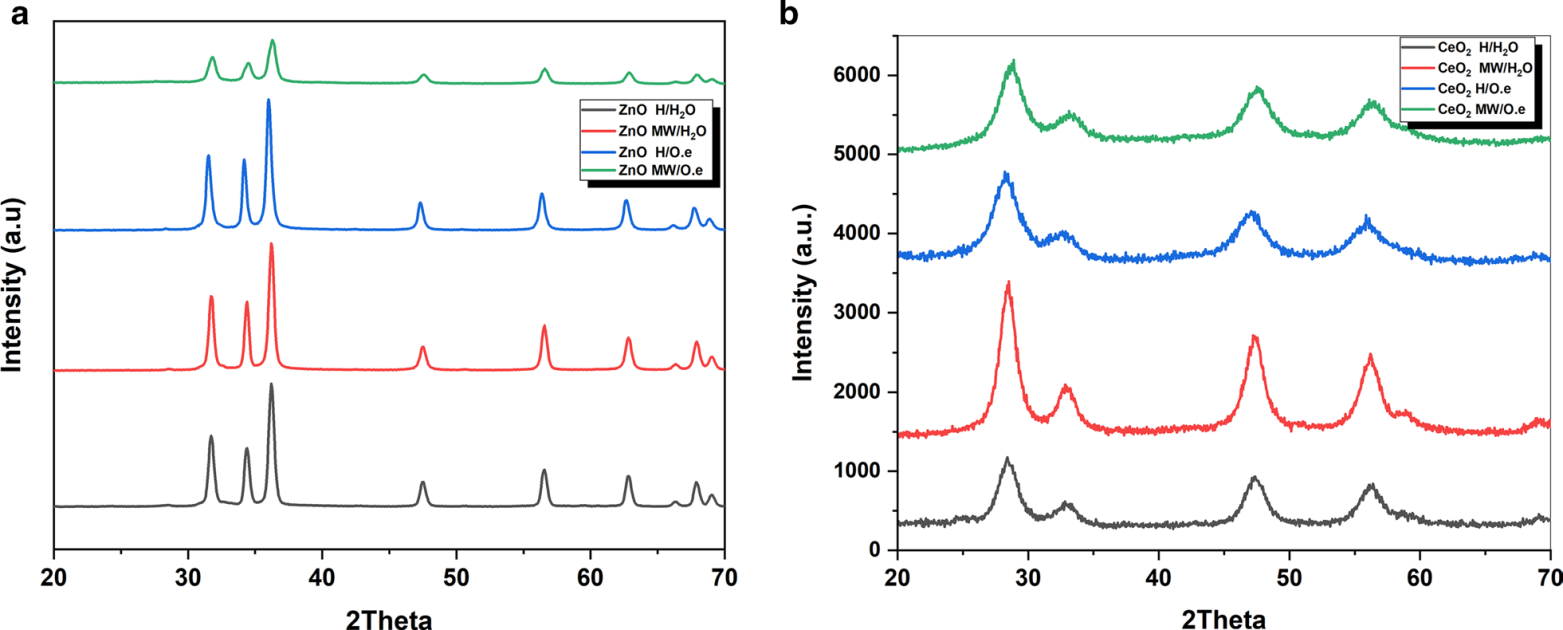
XRD patterns of **a** ZnO and **b** CeO_2_ nanoproducts prepared via microwave-assisted hydrothermal methods compared with traditional hydrothermal methods in the presence and absence of *Olea* leaf extract as a green capping agent

The crystal structure of the green synthesized ZnO and CeO_2_ nanorod structured materials via microwave-assisted hydrothermal methods, compared with that synthesized via traditional hydrothermal methods, was estimated by X-ray diffraction and the representative patterns are given in Fig. 2. All the XRD patterns depicted in Fig. 2a, are indexed with highly crystalline wurtzite-structure from ZnO nanomaterials with JCPDS card No. 00-036-145. The diffraction patterns of CeO_2_ nanomaterials given in Fig. 2b are indexed with crystalline CeO_2_ nanomaterials with JCPDS card No.00-34-0394. Using Debye-formula Scherrer's Eq. 1 (Wang et al. 2020), the average crystallite sizes of the obtained nanoproducts are calculated, and the results are shown in Table 1.

1 $$ D \, = \, 0.9\lambda /\beta \, cos \, \theta $$

**Table 1:** Average crystal sizes, surface area and particle size analyses of the different prepared ZnO and CeO_2_ nanoproducts

Sample name	D (Average crystal size) (nm)	FWHM	2Theta	S_BET_ (m^2^g^−1^)	V_t_ (Total pore volume) cm^3^ g^−1^	Average pore diameter (nm)	Particle size × 10^2^ (nm)
ZnO MW/O.e	15.02	0.618	36.274	7.1215	0.0635	35.70	6.73
ZnO MW/H_2_O	19.89	0.467	36.215	5.9285	0.0547	36.92	–
ZnO H /O.e	21.96	0.423	36.018	–	–	–	–
ZnO H/H_2_O	21.51	0.432	36.220	5.6177	0.0622	44.35	–
CeO_2_ MW/O.e	04.83	1.885	28.280	171.14	0.2260	05.28	8.90
CeO_2_ MW/H_2_O	05.15	1.769	28.684	170.96	0.1884	04.41	–
CeO_2_ H/O.e	06.49	1.403	28.449	–	–	–	–
CeO_2_ H/H_2_O	05.65	1.611	28.469	126.84	0.1754	05.53	–

where *D* is the crystallite size, *θ* is the Bragg angle in degree and *β* is the full width at half maximum (FWHM) of the peak, *λ* is the X-ray wavelength of Cu which is 1.5406 Å.

### BET surface area analysis

**Fig. 3:**
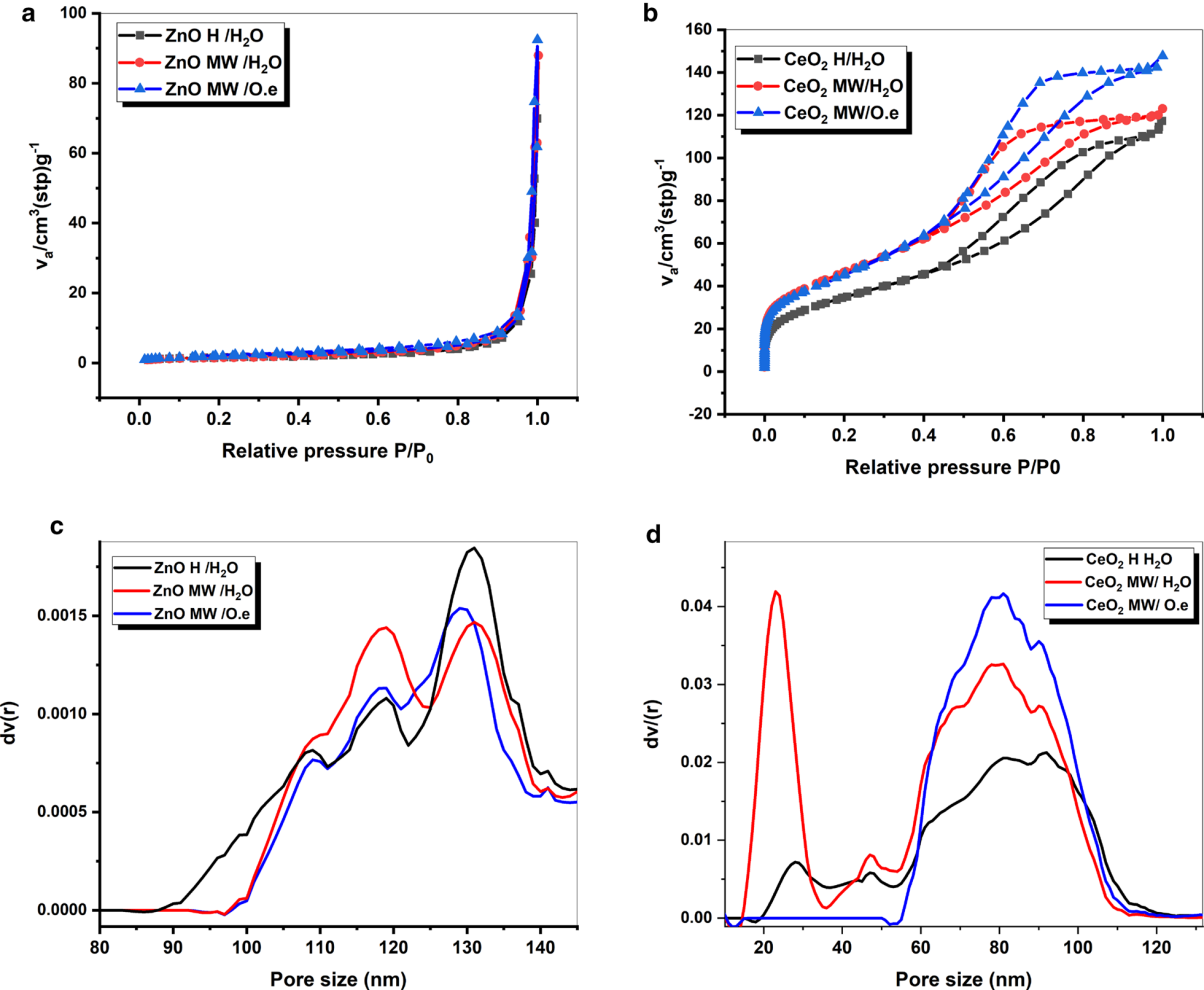
N_2_ isotherms and pore size distribution curves of **a**, **b** ZnO and **c**, **d** CeO_2_ nanoproducts prepared via microwave assisted hydrothermal methods compared with traditional hydrothermal methods in the presence and absence of *Olea* leaf extract as a green capping agent

Using Brunauer–Emmett–Teller (BET) analysis, the porosity and textural characteristics of the green-synthesized ZnO and CeO_2_ nanorod structures are examined. The nitrogen adsorption–desorption isotherms and pore size distribution for ZnO and CeO_2_ nanomaterials prepared by different methods are given in Fig. 3. The BET surface area (S_BET_) and total pore volume of the samples are estimated using the BJH method and are shown in Table 1.

The N_2_-adsorption/desorption isotherms of the different ZnO nanomaterials given in (Fig. 3a), depict a type III with H_3_ hysteresis loop, implying the attendance of macropourous structure. However, the isotherm curves of all CeO_2_ samples (Fig. 3b) posses type-IV isotherms with H_2_ hysteresis loop, implying the attendance of mesoporous structure. It can be well recognized from the surface area results in Table 1, that the green microwave assisted hydrothermal methods (MW/ O.e) leads to increase in the surface area and the porosity of the different nanoproducts.

### Particle size analyzer

The particle size distribution curves of the green-synthesized ZnO and CeO_2_ nanorods measured by the dynamic light scattering (DLS) technique is given in Additional file 1: Fig. S1. From which the average particle sizes of green ZnO and CeO_2_ nanorods are found to be 673 and 890 nm, respectively. On the other hand, the size distribution curve shows that the particle size of both ZnO and CeO_2_ nanorods is polydispersed and larger than the values measured from TEM images.

### FTIR analysis

The FTIR spectra of the green synthesized ZnO and CeO_2_ nanorod structures are displayed in Additional file 1: Fig. S2. The bands were discovered by FTIR analysis at 667.75, 830.24, 1039.47, 1325.83, 1534.64, 1624.32, 2080.85, 2364.88, and 3393.76 cm^−1^ in the range of 4000 cm^−1^ to 400 cm^−1^. The band 3393 cm^−1^ was assigned to the O–H stretching vibration (Alrubaie et al. 2019). Stretching vibration at 1624, 1534 and 1039 cm^−1^ were attributed to C=O, C=C and N–H, respectively. Flavonoids, glycosides, proteins, phenols, and terpenoids with functional groups of alcohols and ketones were found in bioreduction reactions in the bands 3393, 1624, and 1534 cm^−1^.The bands detected at 667 and 427 cm^−1^ confirm the formation of nano-sized ZnO (Alrubaie et al. 2019). The formation of CeO_2_ nanorods indicates by the stretching band of Ce–O which can be seen at 452 cm^−1^.

### Antibacterial activity

**Fig. 4:**
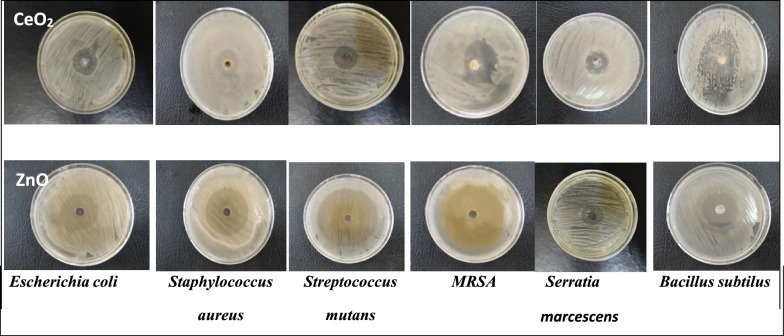
The photographs of the antibacterial test for green synthesized ZnO and CeO_2_ nanorods against six different pathogenic bacterial; *E. coli (ATCC 8739)*, *Serratia marcescen, Staphyllococcus aureus (ATCC 6538), Bacillus subtilis (ATCC 6633), Streptococcus mutant (ATCC 25175)*, and *MRSA*

The photographs of the antibacterial test for green synthesized ZnO (MW/*O.e*.) and CeO_2_ (MW*/O.e*.) nanorods against the six different pathogenic bacterial strains; two gram negative bacteria (*E. coli (ATCC 8739)* and *Serratia marcescens*, and four gram positive pathogens *(Staphyllococcus aureus (ATCC 6538), Bacillus subtilis (ATCC 6633)*, *Streptococcus mutant (ATCC 25175)*, and *MRSA*, are depicted in Fig. 4 in comparison with that prepared with traditional hydrothermal methods with and without *Olea* leaf extract.

**Fig. 5:**
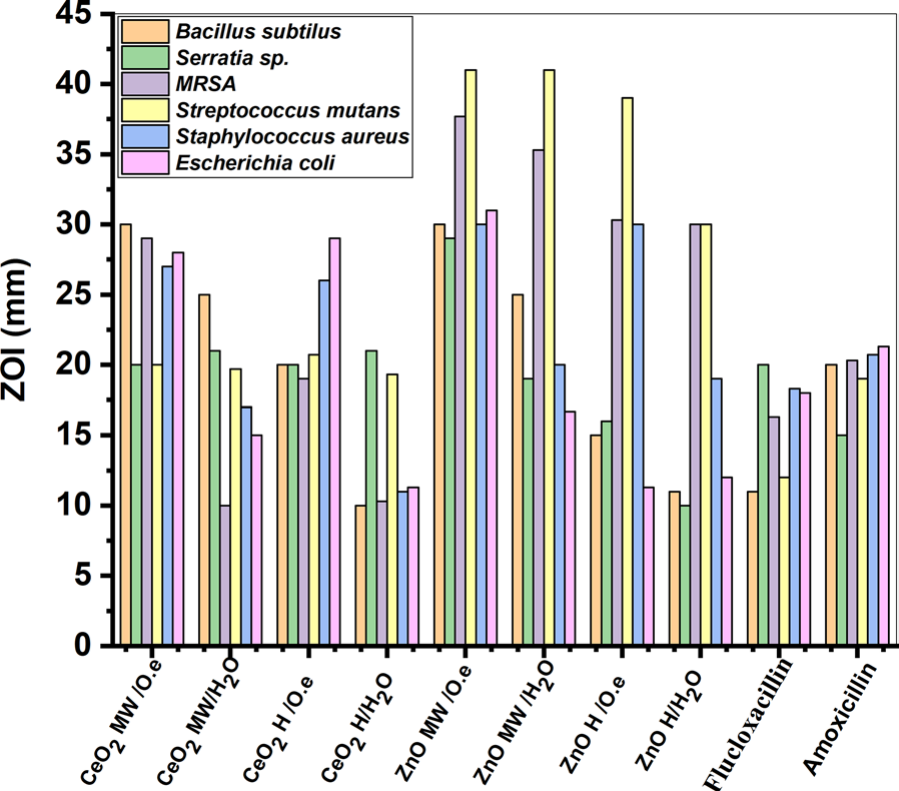
Bar presentation of the antibacterial activity (ZOI; mm ± SD) of the synthesized nanomaterials against the different six pathogenic bacteria

**Scheme 1:**
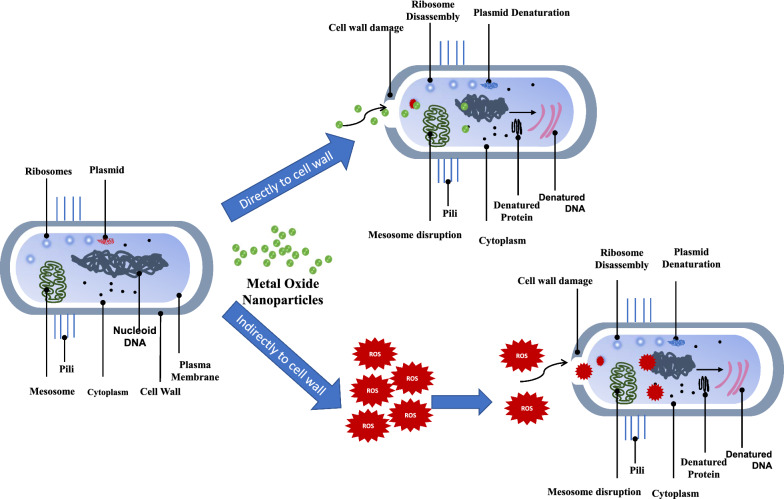
Proposed mechanism of the antibacterial activity of ZnO and CeO_2_ metal oxide nanomaterials including; **a** direct and **b** indirect contact with the cell membrane of the microbe

The antibacterial activity of the green synthesized ZnO and CeO_2_ nanorods investigated against the different pathogenic under investigation was analyzed regarding the zone of inhibition (ZOI) of the bacterial growth and the results are given in Table 2 and presented in Fig. 5. Amoxicillin and Flucloxacillin antibiotics were used as control. Therefore, a proposed mechanism for the antimicrobial effect of the green synthesized ZnO and CeO_2_ nanorods, is presented in Scheme 1.

**Table 2:** The antibacterial activity (ZOI; mm ± SD) of the synthesized nanomaterials against the different six pathogenic bacteria; SD (standard deviation)

Sample name	Zone of Inhibition (ZOI) (mm) Each value is Mean ± SD (n = 3)					
	*Bacillus subtilus*	*Serratia* sp.	*MRSA*	*Streptococcus mutans*	*Staphylococcus aureus*	*Escherichia coli*
CeO_2_ MW/O.e	30.0 ± 1.0	20.0 ± 1.0	29.0 ± 1.2	20.0 ± 1.5	27.0 ± 1.0	28.0 ± 1.0
CeO_2_ MW/H_2_O	25.7 ± 0.6	21.0 ± 1.0	10.0 ± 1.0	19.7 ± 1.2	17.0 ± 1.0	15.0 ± 1.0
CeO_2_ H /O.e	20.0 ± 1.0	20.0 ± 1.0	19.0 ± 1.0	20.7 ± 1.5	26.0 ± 1.0	29.0 ± 1.0
CeO_2_ H /H_2_O	10.0 ± 1.0	21.0 ± 1.0	10.3 ± 0.6	19.3 ± 0.6	11.0 ± 1.0	11.3 ± 0.6
ZnO MW/O.e	30.0 ± 1.0	29.0 ± 1.0	37.7 ± 0.6	41.0 ± 1.0	30.0 ± 1.0	31.0 ± 1.0
ZnO MW/H_2_O	25.7 ± 0.6	19.0 ± 1.0	35.3 ± 0.6	41.0 ± 1.0	20.0 ± 1.0	16.7 ± 0.6
ZnO H /O.e	15.0 ± 1.0	16.0 ± 1.0	30.3 ± 1.2	39.0 ± 1.0	30.0 ± 1.0	11.3 ± 0.6
ZnO H /H_2_O	11.0 ± 1.0	10.0 ± 1.0	30.0 ± 1.0	30.0 ± 1.0	19.0 ± 1.0	12.0 ± 1.0
Flucloxacillin	11.0 ± 1.0	20.0 ± 1.0	16.3 ± 0.6	12.0 ± 1.0	18.3 ± 0.6	18.0 ± 1.0
Amoxicillin	20.0 ± 1.0	15.3 ± 0.6	20.3 ± 1.5	19.0 ± 1.0	20.7 ± 0.6	21.3 ± 0.6

### Cytotoxicity and antitumor activity

**Fig. 6:**
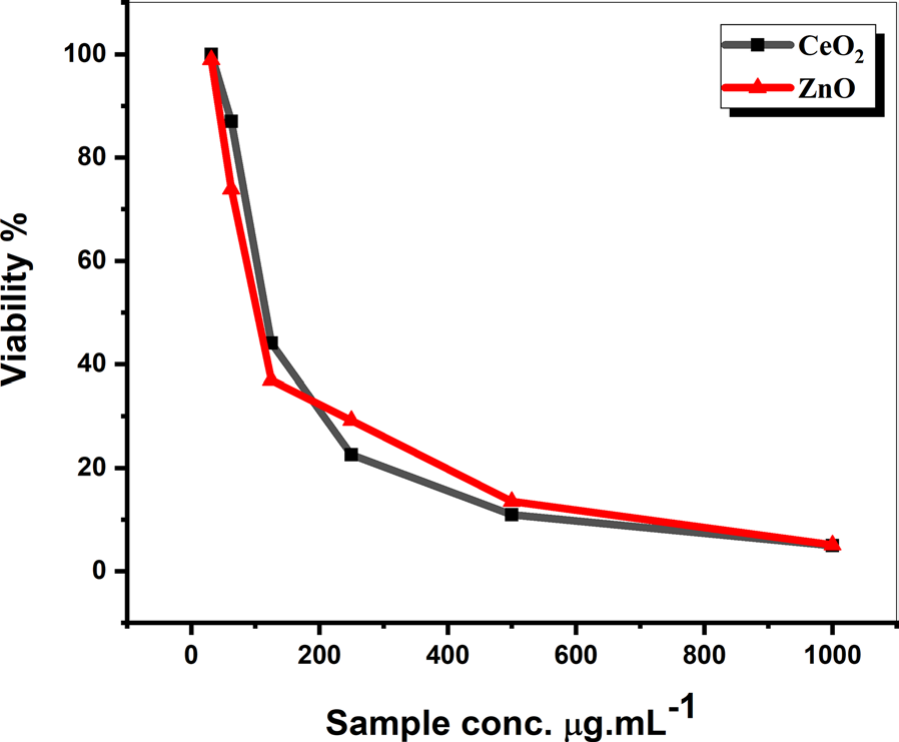
The cell viability curves of *in-vitro* cytotoxic effect of ZnO and CeO_2_ nanorods green synthesized via microwave-assisted hydrothermal method in the presence of *Olea* leaf extract as capping agent on the hepatocellular carcinoma (HeG2) cell lines

The antitumor activities of the green-prepared ZnO and CeO_2_ nanorods via microwave-assisted hydrothermal methods using *Olea* leaf extract as a capping agent are tested on hepatocellular carcinoma cell lines. The results are investigated in terms of the cell viability curves (Fig. 6) and identified with IC_50_ values which were 103.5 and 117.24 µg mL^−1^ for the green synthesizes ZnO and CeO_2_ nanorod structures, respectively.

**Fig. 7:**
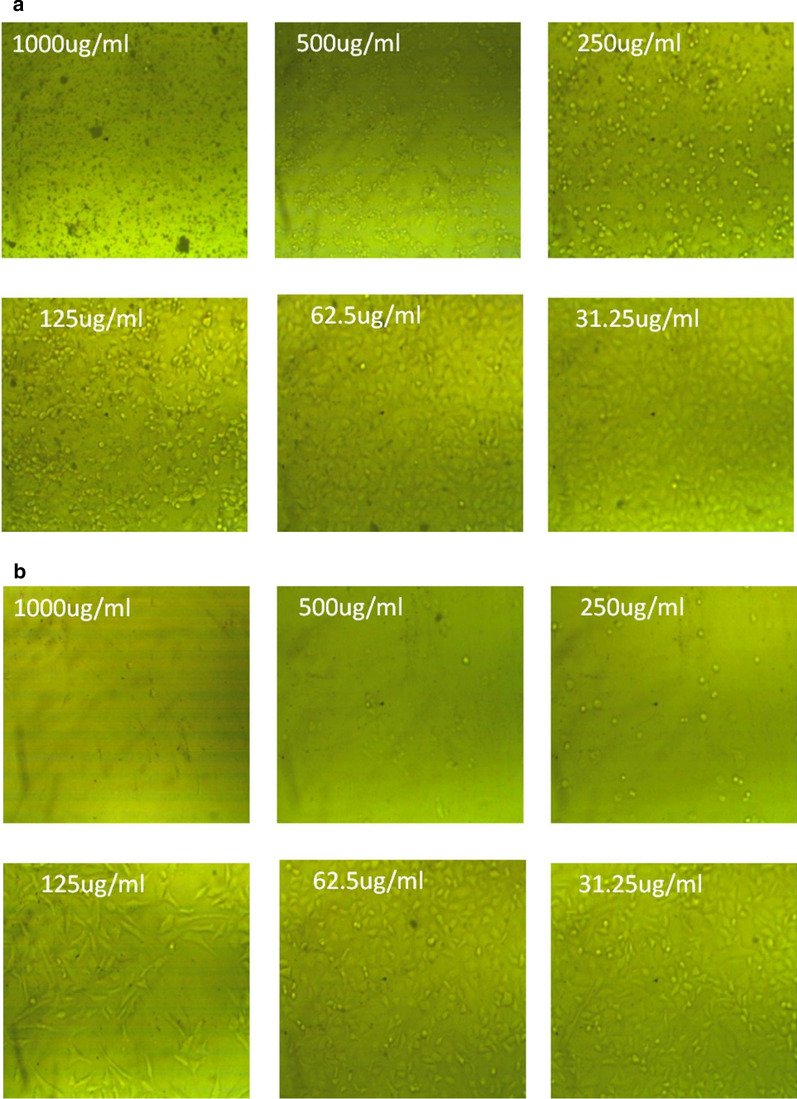
Inverted microscopy images of the cytotoxic effect of the green synthesized **a** ZnO and **b** CeO_2_ nanorods via microwave-assisted hydrothermal method using *Olea* leaf extract as capping agent by crystal violet staining on Hepatocellular carcinoma (HeG2) cells

The Inverted microscopy images of the cytotoxic effects of different concentrations from the green synthesized ZnO and CeO_2_ nanorods on Hepatocellular carcinoma (HeG2) cells, are given in Fig. 7 a and b, respectively.

### In-vivo antitumor toxicity

The in-vivo toxicity test (LD_50_) for the green-produced ZnO and CeO_2_ nanorod structures was performed on mice groups that received a 0.1–3 g kg^−1^ oral gradual increasing dose of the investigated nanomaterial. The findings revealed that neither morbidity nor mortality was observed. Whereas, the above doses didnot not cause any acute poisoning symptoms. This reveals that even at high concentrations, the green-synthesized ZnO and CeO_2_ nanorods are non-toxic (LD_50_ higher than 3000 mg kg^−1^). Moreover, the toxicity and cytotoxic activities of the green-synthesized ZnO and CeO_2_ nanorods via microwave-assisted hydrothermal method using *Olea europaea* leaf extract have been first reported against Hepatocellular carcinoma (HeG2) cells.

## Discussion

Highly crystalline ZnO and CeO_2_, nanorod structured materials have been prepared via microwave-assisted hydrothermal methods based on *Olea*. *europaea* leaf plant extract as a capping agent and compared with traditional hydrothermal methods. The surface morphology results from the TEM images (Fig. 1) of the green synthesized samples confirm the shape-controlling effect of the green extract during the microwave irradiation synthesis process. (Xiaoyan et al. 2014). Moreover, the textural analysis from XRD results given in Table 1, proved that the presence of *O.e*. leaf extract as a capping agent leads to reduction of the average crystallite size of the produced nanostructured materials in comparison with the traditional hydrothermal methods. This demonstrates that microwave-assisted hydrothermal methods, in synthesis of nanomaterials, is better than traditional hydrothermal methods and green chemistry approach is a novel and eco-friendly method that is also better than traditional chemical method in controlling the size and morphology of the produced nanomaterials (Wason et al. 2013). The proposed mechanism for the effect of *Olea europaea (O. e.)* leaf plant extract as a natural medicinal capping agent is depicted in the representative scheme in Additional file 1: Fig. S3. In this representation mechanism, the aqueous extract of *O. europaea* includes a series of compounds that might be effectively utilized as chelating agents. The Oleuropein (the active material) can be easily dissociated into Oleanolic acid and Hydroxytyrosol. The latter is a powerful reducing agent that makes chelation around Zn^2+^ or Ce^4+^ to form a chelation ring. This enables controlling the size and shape of the growing rods from ZnO and/or CeO_2_ during the microwave hydrothermal synthesis process (Maqbool et al. 2016).

From the surface area results given in Table 1., the specific surface area (S_BET_) and the total pore volume (V_t_) are increased remarkably increased by applying the green synthesis route using *O.e*. leaf extract, as a green capping agent. According to the literature reported by Naidi et al. (2021), the high surface area and porous nature of the nanomaterial leads to increase the reactive oxygen species on the surface and gives good contact with the microbes and hence enhances its antimicrobial effect (Naidi et al. 2021).

Moreover, the decrease in the average particle sizes of both ZnO and CeO_2_ nanorods measured by DLS technique may also promote the formation of oxygen vacancies which will result in higher antimicrobial and antitumor activity (Faisal et al. 2021).

The green synthesized ZnO and CeO_2_ nanorods exhibited an effective control for infectious diseases and antibacterial activity against both G+ve and G−ve pathogens when compared with either traditional synthesized nanoparticles or standard antibiotic control drugs such as Amoxicillin and Flucloxacillin antibiotics. This can be concluded to the ZnO and CeO_2_ NRs synthesized via green chemistry have great potential for future antimicrobial therapy.

From the results given in Table 2 and the selected images presented in Fig. 5 prove the effective antimicrobial activity of the green synthesized CeO_2_, and ZnO NRs via microwave-assisted hydrothermal methods using *Olea* leaf extract as capping agent compared with the traditional hydrothermal methods. This demonstrated that green chemistry and microwave are more effective method for synthesis of CeO_2_, and ZnO NRs that have antimicrobial application better than traditional methods and better than control antibiotics (Zhang et al. 2019). This could be attributed to increasing the surface area and the porosity of the obtained nanorod structures. As the high surface area and porous nature of the nanomaterial leads to increase the reactive oxygen species on the surface and gives good contact with the microbes and hence enhances its antimicrobial effect and control infectious diseases (Lalabadi et al. 2019).

Furthermore, the observed difference in ZOI among different bacterial strains under examination could be attributed to unique bacterial cell wall shape, which aids pathogens in resisting against the applied nanomaterial. Other properties, such as the rate of applied nanomaterial diffusion across the bacterial envelope, ionic discharge, binding activation energy, chemical properties, and surface charge attraction, are also play important role against a variety of bacterial infections. Gram-negative pathogens also have different cell wall structural features, such as the deposition of lipopolysaccharide material, which increases their pathogenicity.

Additionally, it has been shown that when compared to NPs made using other physical or chemical methods, green manufactured nanorods have a low level of genotoxic and cytotoxic nature for normal somatic cells with proven efficacy (Maqbool et al. 2018). Also, the smaller particle size of the green synthesized nanorods, makes it across the cellular membrane of the bacteria. This explains its higher bacterial activity than the nanoparticles synthesized by traditional hydrothermal methods (Foster et al. 2010). Although the antimicrobial activities of ZnO and CeO_2_ NPs have been reported in several studies, the exact mechanism has not yet been elucidated (Krishnamoorthy et al. 2014).

The statistical results represented in Fig. 5 show that by comparison with the nanoproducts prepared either by traditional hydrothermal methods (ZnO H/H_2_O, ZnO/H *O.e*. and CeO_2_ H/H_2_O, CeO_2_ H/*O.e*.) or by microwave synthesis methods without addition of *O. europaea* (ZnO MW/H_2_O and CeO_2_ MW/H_2_O), the green synthesized ZnO MW/*O.e*. and CeO_2_ MW/*O.e* nanorod structures synthesized via microwave-assisted hydrothermal methods leads to larger zone of inhibition against all examined bacterial strains.

The proposed mechanism given in Scheme 1 suggested antimicrobial route of the green synthesized ZnO and CeO_2_. Which represented that the metal oxide nanomaterial may make direct contact with the cell membrane of the microorganism and damages the cell wall and penetrates inside the cell then generates reactive oxygen species (ROS) which may affect the DNA (ribosomes, and/or proteins).

The second suggested pathway is indirect contact, which caused the NPs to interact with the bacterial environment outside the cell and produce ROS. ROS then enters the cell through the damaged cell wall and also disrupts DNA, ribosomes, and proteins in addition to disrupting proteins, ribosomes, and other biochemical processes. Both mechanisms eventually result in cell death (Abdo et al. 2021; Alahmdi et al. 2022).

The Inverted microscopy images of the cytotoxic effects of different concentrations from the green synthesized ZnO and CeO_2_ nanorods on Hepatocellular carcinoma (HeG2) cells clearly show the alterations in the morphology and reductions in tumor cell number indicating the high activity of green synthesized nanorods. These nanorods selectively enlarged the oxidative stress and apoptosis in irradiated cancer cells, while protecting normal tissues (Thakur et al. 2019). They are also exhibited profound anticancer potential. It provides cytoprotection towards healthy cells and kills cancer cells through encouraging reactive oxidizing species (ROS) formation.

The higher anticancer capability of ZnO nanorods than CeO_2_ nanorods may be related to the smallest particle size of the former according to the DLS results (Nguyen et al. 2019).

So from this research result, the green synthesized ZnO and CeO_2_ NRs revealed a strong and promising antitumor activity against Hepatocellular carcinoma (HeG2) cells. Additionally, the toxicity assay of the green synthesized nanorods proves that even at high concentrations, the green-synthesized ZnO and CeO_2_ nanorods are non-toxic (LD_50_ > 3000 mg kg^−1^).

## Supplementary Information

**Additional file 1: Figure S1.** The particle size distribution curves of the green-synthesized ZnO and CeO2 nanoods measured by the dynamic light scattering (DLS) technique. **Figure S2.** The FTIR spectra of the green synthesized ZnO and CeO2 nanorod structures. **Figure S3.** Mechanism of *Olea europaea* extract as capping agent and role of phyto reductants around Zinc oxide and cerium oxide NRs.

## Data Availability

All data are fully available without restriction.

## Abbreviations

MW/*O.e*: Microwave-assisted synthesis in the presence of *Olea* leaf extract as a green capping agent
MW/H_2_O: Microwave-assisted synthesis in the presence of water
H/O.e: Traditional hydrothermal synthesis method in the presence of *Olea* leaf extract as a green capping agent
MW/H_2_O: Traditional hydrothermal synthesis method in the presence of water
NRs: Nanorods
ZOI: The zone of inhibition
IC_50_: Inhibition concentration
LD_50_: Lethal dose at which 50% of the animals are died
XRD: X-ray diffraction
TEM: Transmission Electron Microscopy
BET: Specific surface area according to (Brunauer, Emmett, and Teller) theory
BJH: Barrett–Joyner–Halendamethod
ZS: Zeta Sizer Nano
DLS: Dynamic light scattering technique
SD: Standard Deviation
