# Supplementary material for: Green microwave synthesis of ZnO and CeO2 nanorods for infectious diseases control and biomedical applications
Source: AMB Express. 2022 Dec 12;12:153. doi: 10.1186/s13568-022-01495-7 (PMC9742012; doi:10.1186/s13568-022-01495-7)
Supplement: Supplementary file 1 — Additional file 1: Figure S1. The particle size distribution curves of the green-synthesized ZnO and CeO2 nanoods measured by the dynamic light scattering (DLS) technique. Figure S2. The FTIR spectra of the green synthesized ZnO and CeO2 nanorod structures. Figure S3. Mechanism of Olea europaea extract as capping agent and role of phyto reductants around Zinc oxide and cerium oxide NRs. [file 13568_2022_1495_MOESM1_ESM.pdf]

Green Microwave Synthesis of ZnO and CeO_2 Nanorods for Infectious Diseases Control and Biomedical Applications

Nevein Gharbia*[1], Sabha Elsabbagh[2], Ali Saleh[3] , Hoda Hafez[4]

[1]Environmental Studies and Research Institute (ESRI), University of Sadat City, Sadat City, 23897 Menofia, Egypt.
[2]Department of Microbiology, Faculty of Science, Menofia University, Menofia, Egypt.
[3]Environmental Geology Lab. Survey of Natural resources, Environmental Studies and Research Institute (ESRI), University of Sadat City, Sadat City, 23897 Menofia, Egypt.
[4]Nano Photochemistry Laboratory, Nanotech. Dept., Environmental Studies and Research Institute (ESRI), University of Sadat City, Sadat City, 23897 Menofia, Egypt.

AMB EXPRESS JOURNAL

Corresponding author: Nevein Gharbia, Environmental Studies and Research Institute (ESRI), University of Sadat City, Menofia, Egypt. P.O. 32897

. Email: neveen.mahmoud@esri.usc.edu.eg

Orchid Number: 0000-0001-6467-5697

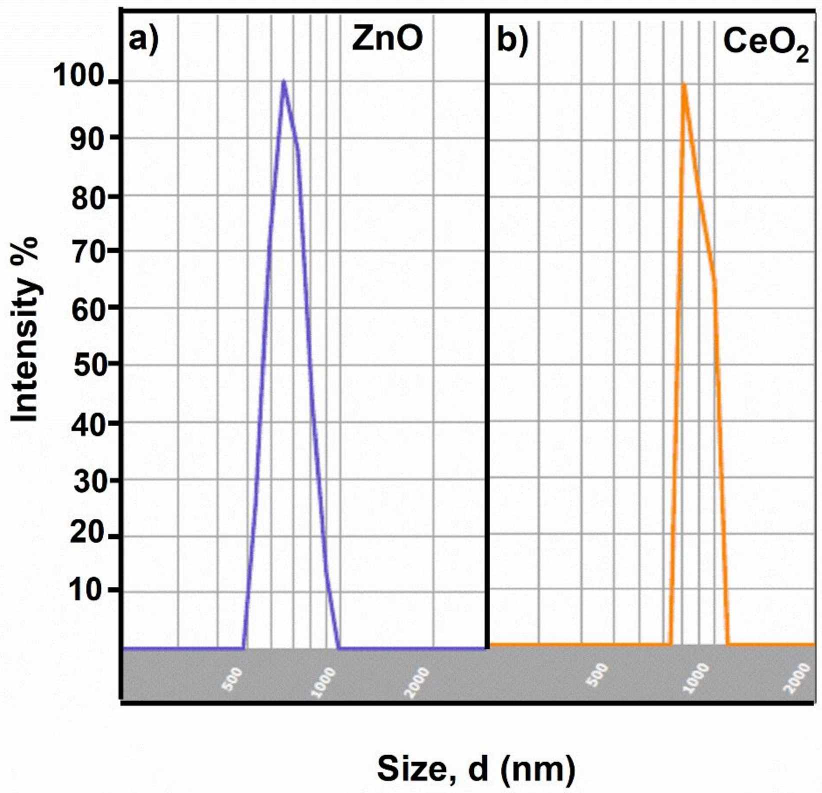

Figure S1: The particle size distribution curves of the green-synthesized ZnO and CeO_2 nanoods measured by the dynamic light scattering (DLS) technique.

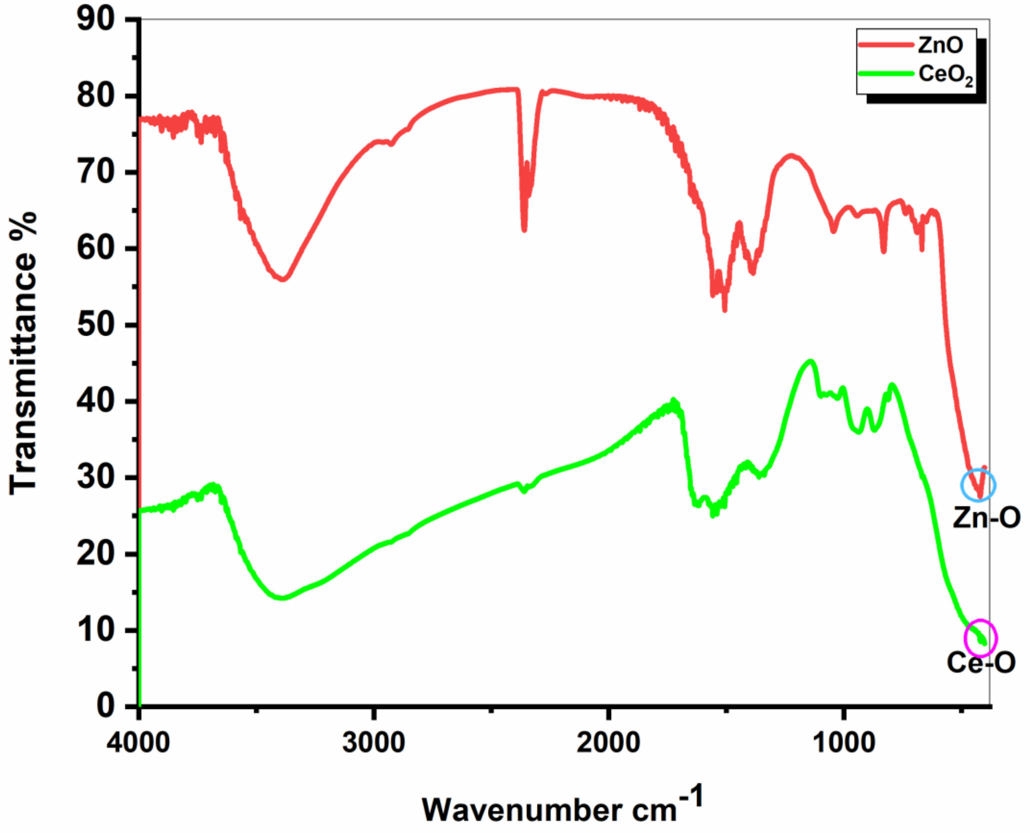

Figure S2: The FTIR spectra of the green synthesized ZnO and CeO_2 nanorod structures.

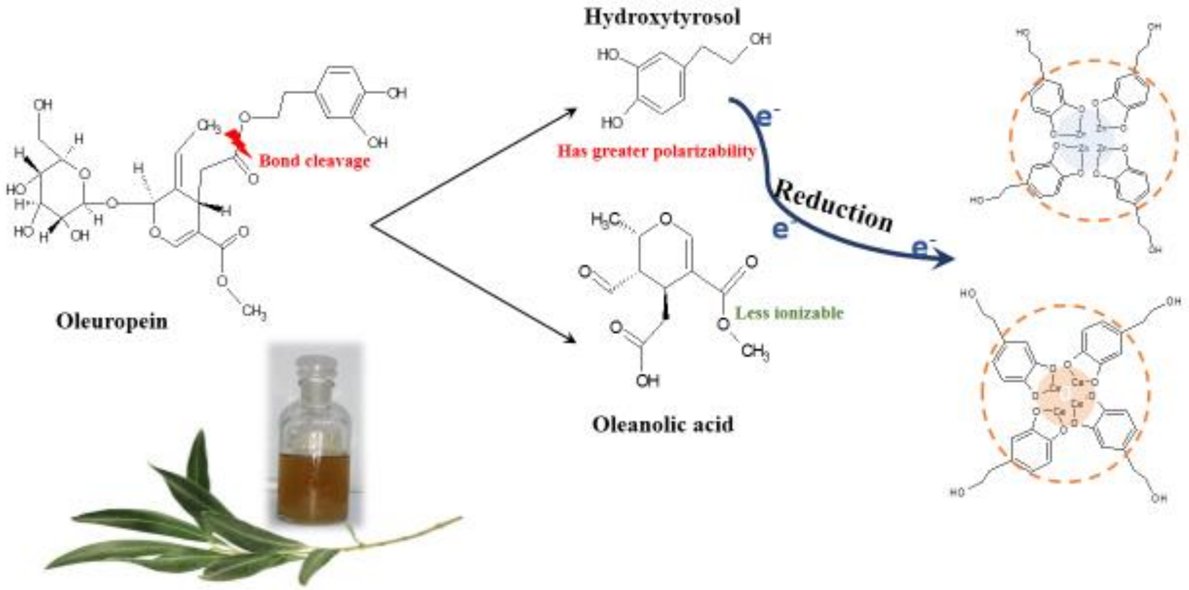

Supplement 3. Mechanism of *Olea. europaea* extract as a capping agent and role of phyto reductants around Zinc oxide and Cerium oxide NRs.

Figure S3: Mechanism of *Olea europaea* extract as capping agent and role of phyto reductants around Zinc oxide and cerium oxide NRs
